# Chronic haemothorax: an important cause of pleural effusion

**DOI:** 10.1002/rcr2.758

**Published:** 2021-05-04

**Authors:** David K. Z. Ching, Edward T. H. Fysh

**Affiliations:** ^1^ Department of Respiratory Medicine St John of God Midland Hospitals Midland WA Australia; ^2^ Department of Intensive Care St John of God Midland Hospitals Midland WA Australia; ^3^ Faculty of Health and Medical Sciences University of Western Australia Perth WA Australia

**Keywords:** Anticoagulation, chronic haemothorax, haemothorax, NOAC, pleural effusion

## Abstract

We describe a case of chronic exudative pleural effusion in a patient initially referred with anorexia, weight loss, and past history of breast cancer, following multiple presentations with chest pain and dyspnoea. Detailed history included past blunt thoracic trauma with pleural effusion drainage and anticoagulation for atrial fibrillation (AF). This case highlights several learning points for physicians around the management of thoracic trauma, anticoagulation for AF, and chronic haemothorax as an uncommon but important cause of exudative pleural effusion.

## Graphical abstract

Chronic haemothorax is an uncommon cause of unilateral pleural effusion, and is a potential complication of long‐term anticoagulation therapy.

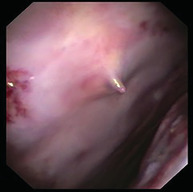

## Introduction

Chronic haemothorax is an uncommon cause of unilateral, exudative pleural effusion, and requires careful diagnostic evaluation. It is a potential complication of long‐term anticoagulation therapy, especially following thoracic trauma. This case highlights the need for careful clinical assessment and monitoring of anticoagulant use in the trauma patient.

## Case Report

A 75‐year‐old lady presented with left‐sided chest pain and dyspnoea after a fall, hitting her chest. Her background included atrial fibrillation (AF), anticoagulated with apixaban (CHA2DS2‐VASc 2), hypertension, and mastectomy for breast cancer 10 years prior.

Assessment revealed rib fractures and a small left hydrothorax. She was admitted under gerontology for pain control and rehabilitation at a secondary teaching hospital. Increasing pain and dyspnoea over five days prompted repeat chest radiography, which showed an increasing pleural effusion. Her apixaban was withheld on day 5 and a 12‐Fr intercostal catheter was inserted. Pleural fluid analysis confirmed a haemothorax (pleural haemoglobin 73, protein 42 g/L, lactate dehydrogenase (LDH) 374 U/L, peripheral blood haemoglobin 96). Computed tomography (CT) chest did not show active contrast extravasation. The haemothorax drained completely over three days and the patient stabilized and was discharged home. Apixaban was withheld, pending outpatient review.

Apixaban was restarted two weeks after discharge. Over the next four months, she developed anorexia and 16 kg weight loss, with increasing dyspnoea and left‐sided pleuritic chest pain. She then re‐presented to hospital following a syncopal event. CT chest showed a recurrent left pleural effusion, a pleural mass, and displaced left eighth rib fracture. Her apixaban was withheld again, and the patient underwent thoracocentesis, which showed a haemorrhagic effusion and no evidence of malignancy or infection as a cause for her constitutional symptoms. She was given instructions to continue withholding apixaban and was commenced on aspirin 100 mg. Given the reported pleural mass and weight loss, she was referred to the respiratory service for pleuroscopic biopsy.

**Figure 1:**
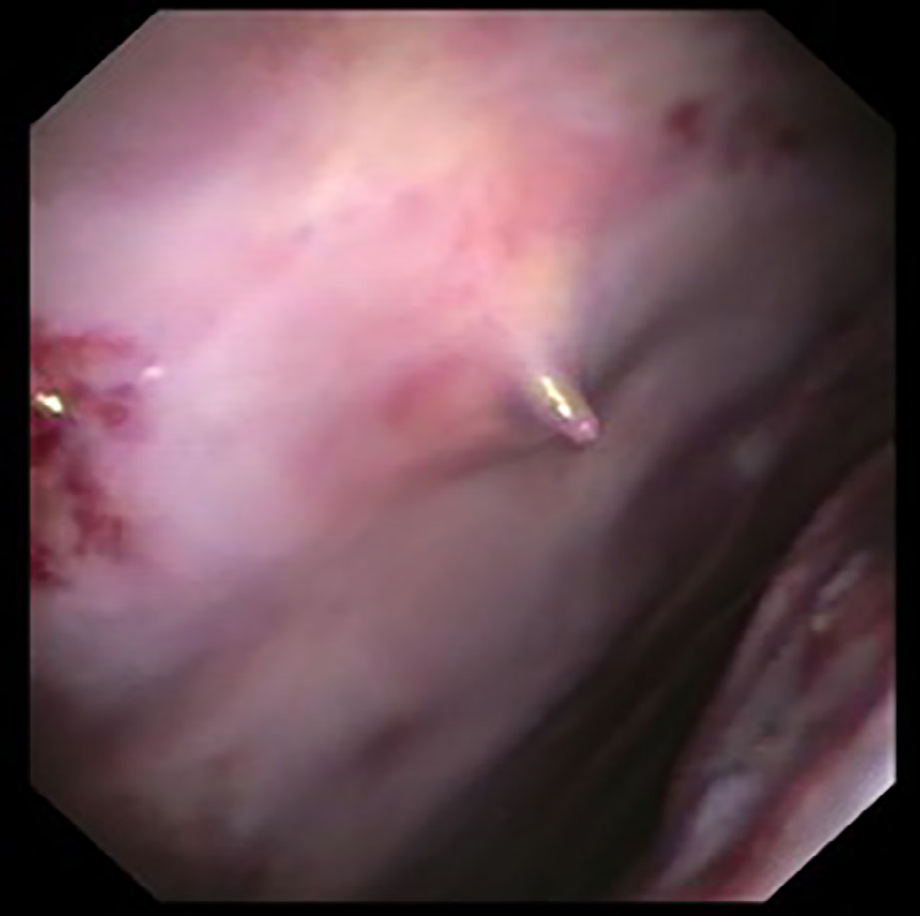
Bone shard from the ninth rib protruding into pleural space.

**Figure 2:**
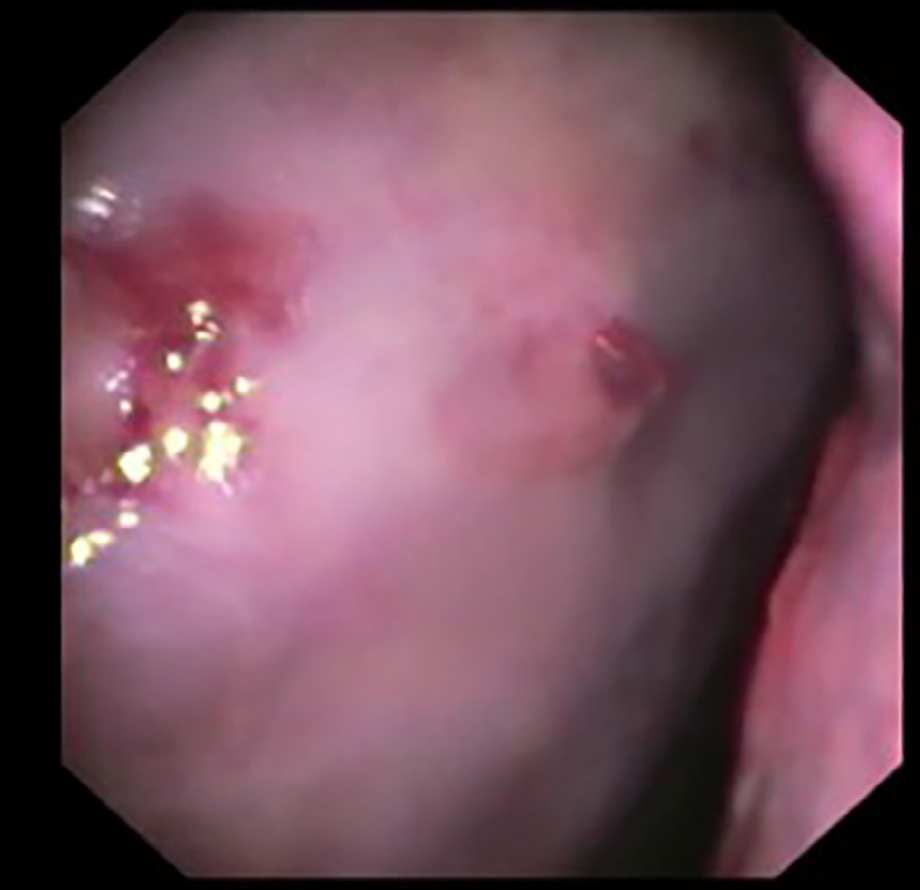
After pleuroscopic resection of bone shard.

Pleuroscopy showed persistent haemorrhagic effusion and clot, left lung laceration, and a 2‐cm rib fragment protruding through the parietal pleura (Fig. 1). The pleura showed diffuse, smooth thickening. The rib fragment was resected endoscopically using forceps via the flexi‐rigid pleuroscope (Fig. 2). Pleural biopsies showed benign chronic inflammation with haemosiderin‐laden macrophages. Microbiological testing including acid‐fast bacilli culture was negative. Cardiothoracic surgeons were consulted regarding possible video‐assisted thoracoscopic surgery (VATS) with fixation of the rib fractures; however, two weeks later, the patient had improved significantly, with bedside thoracic ultrasound showing minimal left pleural effusion.

One month later, the effusion had completely resolved. Repeat CT chest showed callus formation around the rib fractures and no evidence of residual intrapleural fragments. The patient was recommenced on apixaban, with no further complications.

## Discussion

Anticoagulant therapy to prevent thromboembolism in AF is well established and increasing numbers of patients are prescribed non‐vitamin K antagonist oral anticoagulants (NOACs). Evidence to guide NOAC use after trauma is slowly accumulating. There is evidence that restarting NOACs within 90 days of trauma reduces all‐cause mortality (hazard ratio (HR) 0.55) and ischaemic stroke (HR 0.54), but with significant risk of major bleeding (HR 1.15), in a major landmark study [1]. The ongoing risk of bleeding needs to be carefully balanced against the risk of ischaemic stroke, and close monitoring is required when reinitiating oral anticoagulant therapy.

Acute traumatic haemothorax is thought to occur in 300,000 cases per year in the United States. Urgent drainage and careful clearance of residual clot are indicated [2]. On the other hand, chronic haemothorax is one of the less common causes of exudative unilateral pleural effusion, and often requires careful clinical assessment to distinguish it from more common causes such as infection and malignancy [3]. Evidence to guide re‐initiation of NOAC after traumatic haemothorax is lacking.

This case highlights the importance of diligent management of haemothorax in the acute phase and careful consideration of chronic haemothorax at a later date. The case also emphasizes the need for ongoing, experienced monitoring of NOAC use in patients suffering trauma.

In conclusion, the learning points are:

1. Cessation of NOAC after head trauma is well established [1], but should also apply to all other non‐compressible sites of bleeding. There is minimal evidence to guide restarting in thoracic trauma.
2. Trauma should be managed by experienced trauma teams [4, 5].
3. Assessment of pleural effusion should take note of trauma history as well as medical history. Use of anticoagulants should prompt suspicion of haemothorax.
4. Chronic haemothorax can mimic chronic infection or malignancy, and careful history of chest trauma should be taken [3].
5. Patients who have bled on prophylactic anticoagulation warrant close follow‐up of the risks/benefits of ongoing therapy as recommended in the Chest guidelines [4].

### Disclosure Statement

Appropriate written informed consent was obtained for publication of this case report and accompanying images.

### Author Contribution Statement

This case report was jointly written and edited by Dr. David K. Z. Ching and Dr. Edward T. H. Fysh. Final approval of the report for publishing was done by Dr. Edward T. H. Fysh.
